# Poor mental health is associated with the exacerbation of personal debt problems: A study of debt advice adherence

**DOI:** 10.1177/00207640221083205

**Published:** 2022-03-03

**Authors:** Nicole Andelic, Aidan Feeney

**Affiliations:** 1 University of Aberdeen, UK; 2 Queen’s University Belfast, UK

**Keywords:** Mental health, debt, advice adherence, help-seeking

## Abstract

**Background:**

It is known that there is an association between debt and poor mental health. However, much of the literature is observational and focuses on how debt may lead to poor mental health. Here, we are interested in how poor mental health may be associated with debt advice adherence.

**Aims:**

The aim of the study was to investigate the relationship between mental health and debt advice adherence in individuals applying for a formal debt resolution mechanism (an Individual Voluntary Arrangement, IVA).

**Method:**

Eighty-six participants completed a survey measuring mental health (MHI-5), memory for information discussed during the appointment, attitudes towards IVAs, and trust in the advisor shortly after having a debt advice appointment. Adherence to the advice (whether participants completed the IVA application) was measured 10 weeks later.

**Results:**

The study found that the sample demonstrated poor levels of mental health overall but that non-adherent participants had significantly poorer mental health than those who adhered to the advice.

**Conclusion:**

These results suggest that (a) mental health needs to be considered when advising people with problem debt and (b) future research might examine if mental health support should coincide with important decision points in the debtor’s journey out of debt.

Poor mental health is linked to indebtedness (e.g. Jenkins et al., 2008) and, due to currently high societal levels of personal indebtedness (Money and Pensions Service, 2019; The Money Charity, 2021), debt is likely to feature in the experiences of a large proportion of people suffering mental health problems. In this paper, we will be particularly interested in problem debt, that is, debt which cannot be repaid. Although problematic levels of debt can reflect the cumulative effect of sometimes thousands of poor financial decisions, the association between poor mental health and debt tends to be studied at a gross level permitting conclusions about mental health’s association with the state of indebtedness rather than its associations with consequential financial decisions. Here, for the first time, we will examine the association between mental health and one particularly important financial decision: decisions by people with problem debts about whether to adhere to debt advice.

## Debt and mental health

Unsurprisingly, many studies have found an association between debt-related financial stress and psychological stress (Bridges & Disney, 2010; Hamilton et al., 2019; Selenko & Batinic, 2011), but another strand of the research literature is focussed on the link between debt and poor mental health. A meta-analysis of studies examining that link by Richardson et al. (2013) demonstrated strong evidence for an association between unsecured debt and mental health. For example, individuals who report having debt within the household have significantly lower well-being than non-debtors (Brown et al., 2005), a higher likelihood of common mental disorders (Meltzer et al., 2013), and are more likely to report symptoms of depression, anxiety and anger (Drentea & Reynolds, 2012). The picture becomes starker when we move beyond the relatively crude distinction between indebtedness and non-indebtedness to compare people who are over-indebted to those who are not. For example, individuals who experience over-indebtedness report lower life satisfaction and emotional wellbeing (Ferreira et al., 2021), and among individuals who have contemplated suicide there is a higher likelihood of having repayment difficulties (Hintikka et al., 1998). Meltzer et al. (2011) grouped debts into three categories; utilities, housing-related debts and shopping-related debts. Those who reported having several types of debts were more likely to report suicidal ideation than those who only reported one debt, echoing the findings of Hintikka et al. (1998).

Although the studies above suggest that debt can contribute to the development of mental health problems (Fitch et al., 2011), like most of the studies in the literature they focus on the state of indebtedness rather than on particular financial decisions which may lead to or exacerbate indebtedness. This means that although we can confidently claim that people with problem debt also suffer from poor mental health, it is impossible to make claims about specific financial decisions and mental health. In the current study we are interested in the correlation between poor mental health and the exacerbation of debt problems. In particular, we will examine whether, once in debt, people with poorer mental health are less likely to decide to follow debt advice.

## Debt advice: Access and adherence

For those who are struggling to repay their debt, advice may be available from government-funded debt advice agencies or from private companies. Informal advice is usually provided by centrally funded organisations and can relate to issues such as negotiation with creditors and budgeting advice. However, for some individuals, such advice may not suffice and they may require advice about formal debt resolution mechanisms instead. Such advice may be provided by state-funded organisations or by private debt resolution companies. Formal debt resolution mechanisms are legally binding plans in which individuals become insolvent and their debt is eventually written off. The most common form of insolvency is bankruptcy which exists in some form in most countries. A second form of insolvency involves the individual paying back as much of their debt as is feasible over a limited time, after which the remaining debt is written off. This is known as Bankruptcy Chapter 13 in the US, and as an Individual Voluntary Arrangement (IVA) in the UK, but can be found in many other countries. Although there are both formal and informal mechanisms to help people resolve problem debt, and advice is often available (but see Money and Pensions Service, 2019, for a discussion of service under-provision in the UK), for at least two reasons the effectiveness of that advice is unclear.

First, problem debtors may not seek debt advice, even when it is available. Debt advice conversations are difficult (Andelic et al., 2019) and admitting to having problem debt is often perceived as stigmatising (Hayes, 2000). Consequently, participants who are in debt are difficult to gain access to, particularly if they are reluctant to even seek debt advice or have already approached advice agencies but were deterred for other reasons. Deterrents include having to queue at the debt advice agency (Dearden et al., 2010; Goode & Waring, 2011), lack of confidence (Buck et al., 2009) or fear of being stigmatised due to being a debtor (Goode & Waring, 2011).

A second problem relates to advisees following the advice that they have received. The Money Advice Service (2017a) estimated that 45% of debtors in the UK did not adhere to all actions agreed with their debt advisor during an initial advice session. Beyond this survey very little work has been done on the extent to which people follow the debt advice they receive or why they might not follow that advice. Unfortunately, even where work has been carried out, the nature of the phenomenon under study leads to problems with participant attrition. For example, in a series of studies, Pleasence et al. (2007) examined the effectiveness of debt advice through longitudinal surveys and 42 qualitative follow-up interviews with people who had reported having difficulties with their debt. In both studies it was found that individuals who had received debt advice indicated an improvement in their financial circumstances which they attributed to receiving debt advice. However, only 35% of the respondents remained in the final data collection phase of the original sample of 176, and the 42 qualitative interviewees came from an original pool of 84 respondents. As the participants who did not find the advice useful may have dropped out of the study, it is very difficult to gauge how accurately these results capture the true effectiveness of debt advice. Certainly, if participant attrition rates are in any way reflective of debt advice adherence, then Pleasance et al.’s studies suggest relatively low rates of adherence to debt advice.

In the small literature on debt advice effectiveness and adherence, the focus has been on whether people follow the advice they receive rather than on why they follow that advice. Thus, we can only speculate as to why people might not follow the advice they receive. One obvious factor is poor mental health. It is highly plausible that, given its association with problem debt, poor mental health will also be associated with lack of advice following, although we are not aware of any studies that address this question directly. Certainly in other contexts, mental health is known to play a role in determining whether advice is followed. For example, WHO estimated in 2003 that among patients with chronic conditions in developed countries, only 50% adhere to their medication regime (Sabaté, 2003) and the problem is worse among those with chronic rather than acute conditions (Cramer et al., 2003; Haynes et al., 2002). A number of studies have found that poor mental health predicts non-adherence. For example, van Servellen et al. (2002) found that hospital anxiety and depression scores were significant predictors of treatment non-adherence among HIV-patients. More generally, meta-analyses (DiMatteo et al., 2002; Grenard et al., 2011) have found that depression is strongly associated with non-adherence. Even in studies where treatment adherence is measured particularly rigorously, anxiety and depression were significant predictors of non-adherence (e.g. Stilley et al., 2004). On the basis of these findings, and the well-established link between problem debt and poor mental health, it is likely that problem debtors with poorer mental health will be less likely to follow the debt advice that they receive.

## The current study

Our study was carried out in collaboration with a financial organisation that provided formal debt repayment plans, IVAs, in the UK. The duration of an IVA is 5 years, after which the remaining debt is written off (The Insolvency Service, 2015). Thus, it is a viable alternative to bankruptcy for individuals who cannot feasibly repay their debt in full. Our collaboration with the financial organisation offered a rare opportunity to carry out a study of individuals seeking debt advice. Participants were interviewed after their debt advice appointments during which they were advised that they were eligible to apply for the repayment plan. Thus, we were able to record whether participants adhered to that advice by making an application within 10 weeks of their appointment, and to use measures collected during the interview to predict participants’ decision-making. We measured mental health and predicted that individuals with better mental health would be more likely to adhere to the debt advice they received.

## Methods

In this study we examined whether poor mental health is correlated with the likelihood of adhering to debt advice. The dependent variable was advice adherence (whether the client completed an application for the legal arrangement within 10 weeks of survey participation). As this was an exploratory study, a range of predictors known to be associated with adherence to treatment for mental health problems were included for control purposes. In particular, because advisee attitudes and beliefs are known to predict treatment adherence in a range of contexts (Gonzalez et al., 2005) we measured participants’ attitudes towards IVAs. We also measured trust in the advisor which is known to predict financial advice following (Lachance & Tang, 2012) and adherence to treatment in mental health care settings (Thompson & McCabe, 2012). Finally, poor mental health is associated with cognitive impairment (e.g. Airaksinen et al., 2005; Epp et al., 2012) meaning that the relationship between mental health and debt advice adherence might be mediated by degree of cognitive impairment. However, cognitive impairment is also known to be associated with financial scarcity (e.g. Mani et al., 2013). To rule out the possibility that failure to follow advice might be the result of difficulties processing that advice, whether due to the effects of poor mental health or financial scarcity on cognitive functioning, we administered a test of participants’ ability to remember information covered during the advice appointment. We also recorded whether the advice appointment took place in person or via telephone, whether the survey was administered over the phone or online, the name of the advisor (to ensure an even spread across the company), their client’s appointment reference number and whether clients had chosen the advice mode themselves.

### Participants

Eighty-six participants (49 face-to-face and 37 telephone clients) participated, 32 of whom participated by telephone and the remainder online. All participants had an initial advice appointment with one of 11 advisors from the collaborating financial organisation and were asked at the end of the appointment if they could be contacted by a researcher to hear more about the study. The participation rate was 35.9%. The first 75 participants were offered a ticket in a prize draw for one of ten £25 vouchers. The remaining 11 participants were offered a £5 voucher instead.

### Measures

Mental health was measured using the Mental Health Inventory-5 (MHI-5, Ware & Sherbourne, 1992), a five-item scale designed to measure mental health over the past 4 weeks which has also been demonstrated to have high reliability (α = .74) (Rumpf et al., 2001). Similar levels of internal consistency were found in the current study (α = .79). Participants responded to statements such as ‘Over the past 4 weeks have you been a very nervous person’ and ‘Over the past 4 weeks have you felt downhearted and blue’ on a 6 point scale from 1 (‘All of the time’) to 6 (‘None of the time’).

Attitudes towards IVAs were measured using three items: ‘I feel confident that an IVA is right for me’, ‘I think that an IVA could help many people in my situation’ and ‘At this moment I am certain that I will proceed with an IVA’. Participants indicated their agreement with the statements on a five-point scale. Cronbach’s alpha showed high levels of internal consistency (α = .87).

Trust was measured using an adapted version of the 17-item Mayer and Davis (1999) Trust scale, which has been found to have moderate to high reliability (α = .69−.94) across a variety of contexts (Kim et al., 2004; Tan & Lim, 2009). Participants rated items such as ‘The advisor is very concerned about my welfare’ on a scale of 1 to 5, 1 meaning ‘I strongly disagree’ and 5 meaning ‘I strongly agree’. A factor analysis revealed that 16 out of 17 items loaded highly and positively (factor loads .54−.86) on a single factor. Discarding the item with low factor loadings resulted in a highly reliable 16-item scale (α = .94).

To confirm that there were no differences in memory recall between the adherence and non-adherence groups, we also administered a memory test. The test consisted of 14 randomly ordered true-or-false statements regarding the legal arrangement, for example ‘You are allowed to take out further credit during an IVA’ or ‘Your credit rating will be affected for 6 years after the IVA approval’. The test had an equal number of true/false items, none of the answers could be deduced from the answer to another item, and all questions concerned information which each advisor must cover in an advice appointment.

### Procedure

Questionnaires were administered by phone or via an e-mail containing a link. Ten weeks after participation in the survey, we determined whether participants had adhered to the advice by applying for an IVA.

This study was part of a bigger research project partially funded by the financial organisation and approved by the Research Ethics Committee at the institution where the study took place.

## Results

The majority of the participants in our sample (69.77%) had applied for an IVA after the 10-week period (see Table 1). The average score on the MHI-5 was 46.33 (out of 100) which is much lower than the averages of 75 to 80 previously reported for the general population (see Hoeymans et al., 2004; Strand et al., 2003). Participants’ average score on the memory test (10.28/14) suggested some difficulties retaining factual information about the arrangement for which they had applied whereas participants scored highly on attitudes towards IVAs and levels of trust in the advisor.

**Table 1.** Descriptive statistics of study variables (*n* = 86).

	Frequency/mean
Adhered to advice	60
MHI-5 score	46.33 (22.48)
Memory test	10.28 (1.89)
Attitudes	4.60 (0.63)
Trust in advisor	4.51 (0.53)

*Note.* Standard deviation in brackets.

### Differences between the adherence/non-adherence group

**Figure 1.**
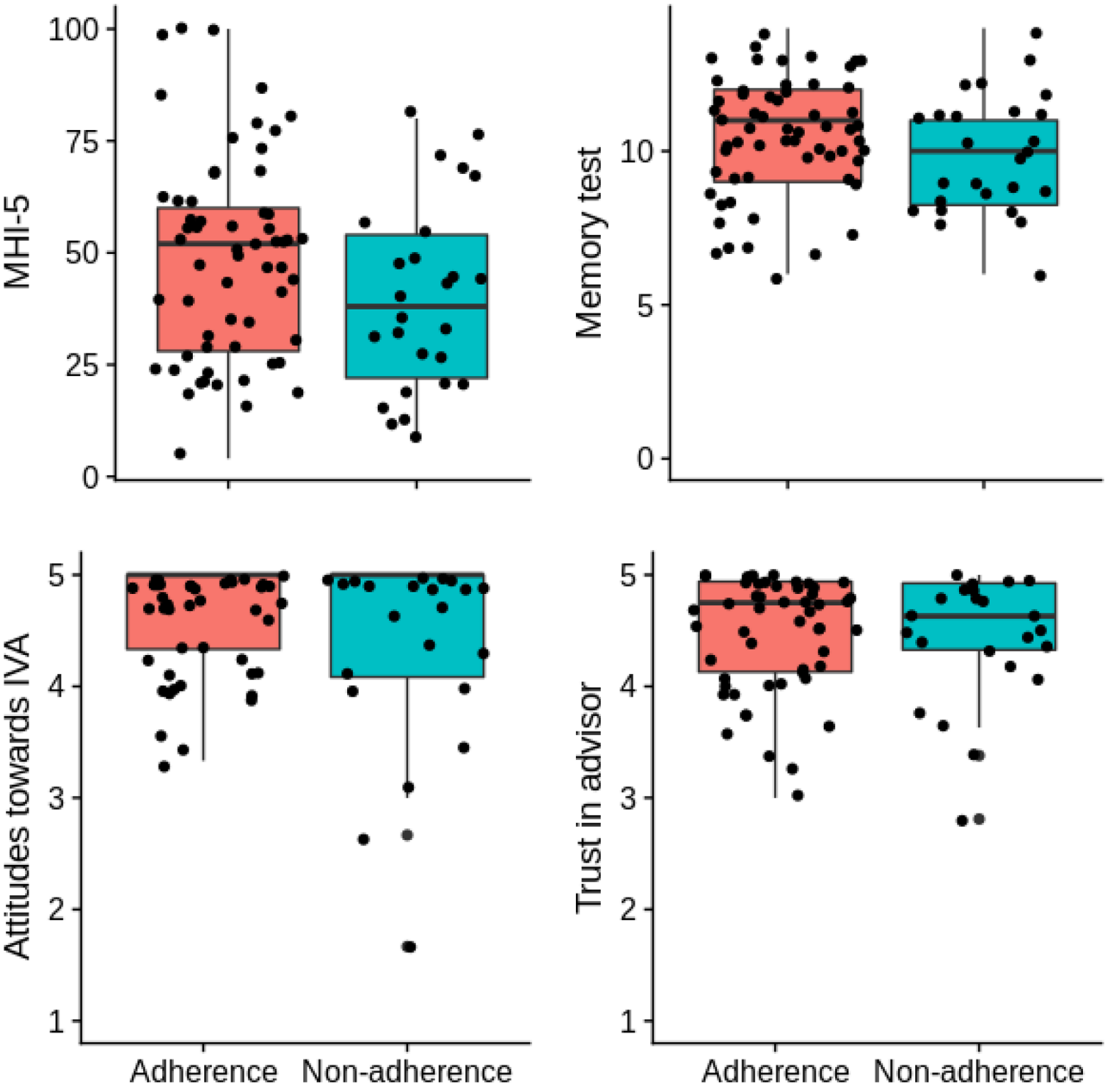
Distribution of mental health, memory test scores, attitudes and trust in advisor broken down by adherence. Jitter applied to reduce overlap.

A one-sided *t*-test showed that non-adherent individuals had significantly lower MHI-5 scores (*M* = 40.00, *SD* = 21.01) than those who adhered (*M* = 49.07, *SD* = 22.71), *t*(84) = −1.74, *p* = .043, *d* = 0.41, 95% CI [UL: −0.39] (see Figure 1). As cognitive difficulties caused by financial difficulties might mediate the relation between poor mental health and non-adherence, we also examined potential differences in number of items recalled from the memory test. Those who adhered to the test recalled on average slightly more items (*M* = 10.45, *SD* = 1.89) than those who did not adhere (*M* = 9.88, *SD* = 1.86), but a one-sided *t*-test revealed this difference to be non-significant, *t*(84) = 1.28, *p* = .101, *d* = 0.30, 95% CI [UL: 0.17]. Furthermore, there was no significant correlation between mental health score and number of items recalled, *r* = −.02, *p* = .832, 95% CI [−0.19, 0.23] A Welch *t*-test for samples with unequal variances found that there were similar attitudes towards IVAs between those who adhered (*M* = 4.67, *SD* = 0.47) and those who did not adhere (*M* = 4.45, *SD* = 0.88), *t*(84) = −1.22, *p* = .116, *d* = 0.31, 95% CI [UL: 0.09]. Similarly, levels of trust in the advisor were equally high in adherers (*M* = 4.52, *SD* = 0.52) and non-adherers (*M* = 4.48, *SD* = 0.56), *t*(84) = −0.30, *p* = .383, *d* = 0.07, 95% CI [UL: 0.17], and trust was not associated with mental health score, *r* = −.14, *p* = .200, 95% CI [−0.07, 0.34].

Interestingly, trust was negatively associated with number of items recalled, *r* = −.25, *p* = .019, 95% CI [0.04, 0.44], such that participants with more trust in the adviser tended to correctly recall less information from the appointment (see Table 2). Although we did not predict this finding it is possible that mistrust in the source of information may lead to more careful processing (see Wang & Bockenholt, 2009).

**Table 2.** Correlations between study variables (*n* = 86).

	MHI-5 score	Memory test	Attitudes
MHI-5 score	–		
Memory test	−0.02	–	
Attitudes	−0.15	−0.05	–
Trust in the advisor	−0.14	−0.25*	0.51***

* *p* < .05. **p < .01. ***p < .001.

A logistic regression showed that the association between adherence and mental health score remained statistically significant (*B* = 0.03, *t*(81) = 1.98, *p* = .048, 95% CI [−0.05, −0.002]) even after including the memory test scores, attitudes and trust in the advisor as control variables. However, perhaps unsurprisingly, a post-hoc power calculation (G*Power 3.1; Faul et al., 2007) showed that the regression is underpowered (55.49%) and so although the results show some evidence for a direct effect of poor mental health on adherence, the logistic regression should be interpreted with caution.

## Discussion

The strong association which is known to exist between indebtedness and mental health is most often considered a consequence of indebtedness causing poor mental health (e.g. Drentea & Reynolds, 2012). By contrast, the aim of the current study was to examine poor mental health as a potential barrier towards debt resolution via its association with adherence to debt advice. We measured mental health shortly after debtors were advised by a debt advisor to apply for a particular debt resolution mechanism and recorded whether they had followed that advice 10 weeks after it was received. We found that non-adherers displayed significantly poorer mental health than adherers, and they continued to do so even when we controlled for attitudes towards the debt resolution mechanism, trust in the advisor and memory for the details of the advice.

Our study makes at least two important contributions to what is known about the relationship between mental health and problem debt. First, our data suggest that, aside from questions about the direction of the causal relationship between poor mental health and the accumulation of problematic levels of debt (see Fitch et al., 2011), poor mental health correlates with difficulties escaping from problem debt. Thus, participants with poorer mental health were significantly less likely to adhere to advice about applying for a legal debt repayment arrangement. Although the correlational nature of our design does not permit a causal claim, we know that participants had poor mental health prior to deciding not to adhere to advice and so we know that poor mental health was not a consequence of non-adherence to this particular piece of advice. Thus, although we cannot rule out the possibility of some other common cause for both poor mental health and decisions not to adhere to debt advice, we can be certain that failure to adhere to advice about the appropriateness of an IVA did not lead to poor mental health. The second contribution made by our study relates to the levels of poor mental health we observed. Although we did not have a control group in this study, we did find levels of mental health that were substantially lower than those that have been reported in studies of the general population (see Hoeymans et al., 2004; Strand et al., 2003). Thus, our results suggest that people with very severe debt problems have substantially poorer mental health than the general population, regardless of whether they adhere to debt advice.

Good mental health is associated with economic well-being (Marmot, 2010). To improve health and well-being in the UK, financial advice has recently been prescribed for people with chronic health conditions (Impact on Urban Health, 2021; Polley et al., 2019), with participants reporting improved confidence and self-esteem after availing of these services (Chatterjee et al., 2018). There is already a large literature on the association between poor mental health and debt. By contrast, our results focus on a specific context where mental health is associated with a particular financial decision related to the exacerbation of debt problems. When giving advice, debt advisors might consider that association and the role it may play in their client’s decision to adhere to the advice they receive.

Although the study design allowed us to follow participants over 10 weeks, the observational nature of our study means that we cannot make causal claims. Furthermore, although we control for some potential confounds there may be other factors which lead to both poor mental health and failure to adhere to advice which we did not measure here. Another potential limitation relates to the size and representativeness of our sample. Due to the nature of the population studied, our sample size was small and, in addition, the participation rate was relatively low. As participants were invited to take part in the survey by their debt advisor, those with higher trust in the advisor may be over-represented in our sample, which may have affected our results in relation to trust in particular. Moreover, although it is clear from our results that people with problem debt have much poorer mental health than the general population, it is not clear that levels of mental health in our sample are representative of levels in the population of people with problem debts. One possibility is that poor mental health acts as a barrier against participation in research (Woodall et al., 2010) and thus problem debtors with extremely poor mental health may be under-represented in our sample. A final limitation is the type of debt advice that is studied here and the degree to which our findings are likely to apply to formal advice only. It is likely that our findings will extend to formal advice given about similar debt resolution mechanisms available in other jurisdictions, although this hypothesis should be tested in future research. However, adhering to informal debt advice may be more difficult than adhering to advice about formal debt resolution mechanisms, as statistics show that formal debt solutions typically have better adherence than informal solutions (Money Advice Service, 2017b). Additional work will be required to examine associations between poor mental health and adherence to informal debt advice.

In conclusion, in addition to the many external factors that can affect a debtor’s ability to resolve their debt problem (Dearden et al., 2010; Goode & Waring, 2011), poor mental health is associated with failure to adhere to formal debt advice. In order to be useful, advice to apply for a formal debt advice mechanism need only be followed once. Accordingly, future studies could examine whether the provision of additional support at the very specific point when people with mental health problems are deciding whether to adhere to debt advice might improve the quality of their decision making and thus, their long term financial prospects.
